# Assessing Canadian women's preferences for cervical cancer screening: A brief report

**DOI:** 10.3389/fpubh.2022.962039

**Published:** 2022-07-28

**Authors:** Patricia Zhu, Ovidiu Tatar, Ben Haward, Gabrielle Griffin-Mathieu, Samara Perez, Laurie Smith, Julia Brotherton, Gina Ogilvie, Zeev Rosberger

**Affiliations:** ^1^Lady Davis Institute for Medical Research, Jewish General Hospital, McGill University, Montreal, QC, Canada; ^2^Department of Psychiatry, McGill University, Montreal, QC, Canada; ^3^Research Center, Centre Hospitalier de l'Université de Montréal (CRCHUM), Montreal, QC, Canada; ^4^Cedars Cancer Center, McGill University Health Center, Montreal, QC, Canada; ^5^BC Women's Hospital, Women's Health Research Institute, Vancouver, BC, Canada; ^6^Melbourne School of Population and Global Health, University of Melbourne, Carlton, VIC, Australia; ^7^Australian Centre for the Prevention of Cervical Cancer, East Melbourne, VIC, Australia; ^8^Departments of Psychology and Oncology, McGill University, Montreal, QC, Canada

**Keywords:** human papillomavirus, cervical cancer screening, cancer prevention, Best-Worst Scaling, HPV test, cytology, women's preferences for cervical cancer screening

## Abstract

Human papillomavirus (HPV) testing is recommended for primary screening for cervical cancer by several health authorities. Several countries that have implemented HPV testing programs have encountered resistance against extended screening intervals and older age of initiation. As Canada prepares to implement HPV testing programs, it is important to understand women's preferences toward cervical cancer screening to ensure a smooth transition. The objective of this study was to assess Canadian women's current preferences toward cervical cancer screening. Using a web-based survey, we recruited underscreened ( > 3 years since last Pap test) and adequately screened (< 3 years since last Pap test) Canadian women aged 21–70 who were biologically female and had a cervix. We used Best-Worst Scaling (BWS) methodology to collect data on women's preferences for different screening methods, screening intervals, and ages of initiation. We used conditional logistic regression to estimate preferences in both subgroups. In both subgroups, women preferred screening every three years compared to every five or ten years, and initiating screening at age 21 compared to age 25 or 30. Adequately screened women (*n* = 503) most preferred co-testing, while underscreened women (n = 524) preferred both co-testing and HPV self-sampling over Pap testing. Regardless of screening status, women preferred shorter screening intervals, an earlier age of initiation, and co-testing. Adequate communication from public health authorities is needed to explain the extended screening intervals and age of initiation to prevent resistance against these changes to cervical cancer screening.

## Introduction

Cervical cancer presents a threat to women's health and the health of all individuals with a cervix. In Canada, each year, approximately 1,300 women are diagnosed with cervical cancer and about 400 die (1). In the last decade, recommendations from multiple health authorities have been updated to include Human Papillomavirus (HPV) testing for primary screening for cervical cancer (2–4). Notably, the World Health Organization (WHO) now recommends HPV testing as the primary method of cervical cancer screening globally (5). Strong evidence exists that HPV testing provides significantly greater protection against invasive cervical carcinomas compared with cytology (Pap test) and earlier detection of cervical pre-cancers (6–8). While recommendations for cytology-based screening typically include a 3-year interval between negative tests, HPV-based screening is recommended at a 5-year interval, considering the test's high negative predictive value (2, 9, 10). Moreover, in countries that have transitioned from cytology-based to HPV-based primary screening programs, the age of screening initiation has changed from 21 to 25 years (Australia, the UK) (4) or 30 years (the Netherlands) (11) considering both the limited evidence of benefit in screening younger women and the harms of over-screening that lead to overtreatment of cervical lesions and subsequent adverse reproductive outcomes (12, 13). The Canadian Partnership Against Cancer Action Plan's goals for elimination of cervical cancer include screening 90% of eligible women with an HPV test (14). Currently, most provinces are preparing to include HPV-testing as the primary approach (including self-sampling, the option of self-collecting vaginal samples for HPV testing) in cervical cancer screening programs.

Important challenges in transitioning from cytology to HPV-based primary screening are due to women's and healthcare professionals' hesitancy to accept longer screening intervals and postpone screening initiation beyond 21 years (15, 16). Implementation experience from Australia and Wales indicate that women have anxieties and concerns about moving to longer screening intervals (every 5 years) and a later initiation of cervical cancer screening (25 years old) which resulted in up to 1.2 million women signing online petitions against the transition toward HPV testing (17, 18). In Canada, findings from the HPV FOCAL trial in British Columbia have presented evidence related to women's acceptance of longer intervals with HPV-based screening and later screening initiation beyond 21 years (19, 20). The HPV FOCAL trial was a 48-month randomized controlled trial comparing the incidence of cervical intraepithelial neoplasia (grade 2 or greater) in participants who received HPV testing for cervical screening vs. cytology. At the 48-month study exit, all participants received co-testing (HPV and cytology) allowing for assessment of acceptance of HPV testing. Given the HPV FOCAL cohort received education about and experience with HPV testing, their preferences toward cervical cancer screening may not be reflective of the Canadian population's baseline attitudes.

As most Canadian women have not yet had experience with HPV testing to date, it is important to understand women's current preferences for cervical cancer screening modalities (cytology vs. HPV testing), age of screening initiation (beyond age 21), and longer screening intervals (5 years or more after a negative HPV test) to inform future implementation strategies. Achieving optimal screening uptake in the context of transitioning from cytology to HPV test-based screening could be complicated by low acceptability for the change among women who are accustomed to existing screening recommendations (21, 22). Additionally, there is a need to examine and address existing barriers to screening amongst vulnerable women (e.g., lower socioeconomic status, recent immigrants) (23, 24). The objective of the study is to assess Canadian women's current preferences for cervical cancer screening in order to develop appropriate communication and education strategies about HPV testing for both currently underscreened and adequately screened women.

## Methods

### Study design and participants

In the present study, we used a cross-sectional design to collect data from Canadian women using a web-based survey in October/November 2021. The study was conducted as part of a larger project funded by the Canadian Institutes of Health Research estimating psychosocial correlates of women's intentions to participate in HPV-based primary screening to prevent cervical cancer. Women answered questions about the following topics: their screening history and health; knowledge of cervical cancer screening; knowledge of HPV testing; attitudes and beliefs toward HPV testing; attitudes and beliefs toward HPV-based self-sampling; and preferences for cervical cancer screening options. Several specific informative statements were provided at different points of the survey to give participants basic information before responding to each section. One informative statement introduced that HPV was the primary cause of cervical cancer, and that HPV testing could allow longer intervals between cervical cancer screenings: “Research shows that if HPV DNA is not found, women are at very low risk for cervical cancer and do not need to screen for cervical cancer as often as with the Pap test (e.g., every 5 years)”. All informative statements are available in Appendix A. Full details of the overall study design and all measures used are available elsewhere, (25).

Participants who met the following criteria were enrolled in the study: (1) Canadian resident; (2) biologically female; (3) between the ages of 21–70; and (4) having a cervix (e.g., never undergone a hysterectomy). The exclusion criterion was having been previously diagnosed with cervical cancer.

Data collection was facilitated by Dynata, and international survey company. Dynata uses a combination of email, website, and in-app invitations to invite participants to complete a survey on “Health and Wellness.” Recruitment included census-based quotas for primary language (English/French) and province of residence to ensure sample representativeness. Oversampling was used to ensure half of the participants were underscreened for cervical cancer (i.e., longer than three years since previous Pap test or never screened). Participants responded to the survey in either English or French. Ethics approval was obtained from the Research Ethics Board of the CIUSSS West-Central Montreal (Project ID: 2021–2632).

### Variables

Socio-demographic variables included both continuous (age) and categorical variables. Some variables were recategorized due to small cell counts. Gender was measured using validated categories that capture socially constructed roles, identities, and behaviors (26), and two categories were retained for analysis: female, and other. Household income was measured in $10,000 increments. Self-reported ethnicity was measured using the nine categories recommended by Statistics Canada (27), and was recategorized into North American Aboriginal, Other North American, European, Asian, and Other (i.e., Caribbean, Latin, Central and South American, African, dual/mixed ethnicities, and uninterpretable open-ended responses). Province or territory was recategorized into Western, Central, and Eastern Canada. Marital status was measured using three categories: married/common-law, single, and dating but do not live with partner. Language spoken at home was measured using three categories: English, French, and other. Dichotomous (yes/no) socio-demographic items included: identification as a visible minority; influence of religious or spiritual beliefs on health decisions; living in Canada for 10 years or more; and completion of a trade certificate/diploma, college or CEGEP degree, or University degree.

Cervical cancer screening history was assessed using the following item: “*When did you have your last Pap test? Select the option that best describes you*.” Response options included: (a) *I had a Pap test within the last year*; (b) *I had a Pap test within the last 1 to 3 years*; (c) *I had a Pap test over 3 years ago*; (d) *I have never had a Pap test*. Participants answering “a” or “b” were categorized as “adequately screened” and those answering “c” or “d” were categorized as “underscreened.”

Unlike traditional methods of assessing preferences (e.g., multiple choice questions), Best-Worst Scaling (BWS) methodology allows for the in-depth assessment of preferences by asking participants to consider the trade-offs of selecting certain combinations of attributes and attribute levels over others through a series of questions that contain a random combination of attribute-levels. We used case 2 BWS methodology (28) for measuring preferences for screening intervals (Domain A) and age of screening initiation (Domain B). Both domains shared the same four attributes (screening methods), which were: Pap test; HPV test; both the Pap test and the HPV test (herein, “co-testing”); and HPV test using self-sampling. For each attribute, we selected three attribute-levels (i.e., screening interval options for Domain A, i.e., every 3 years, every 5 years, every 10 years) and three attribute-levels reflecting age of initiation options for Domain B (i.e., 21 years old, 25 years old, 30 years old). The attribute-levels were chosen based on current cervical cancer screening recommendations or programs in Canada, as well as in other countries where HPV testing has already been implemented (2, 29–31). For each of the two domains, participants answered nine questions, for a total of 18 questions. The order of questions within a domain and the order of the domains were randomized to minimize response bias. To generate questions we used the orthogonal main effect design methodology recommended by Aizaki (32) and the R software packages “DoE.base” (33) and “support.BWS2” (32). See Appendix B for Domain A and Domain B questions.

### Statistical analysis

To identify careless responders, we incorporated two attention check questions in the survey. Participants were instructed to select a specific response choice from a Likert scale (e.g., Please select “strongly agree,” for this question only). Those who responded incorrectly to both attention check questions were excluded from subsequent analyses. Straightliners (i.e., those who responded to all items with the same answer) were identified by calculating their response variance to one of the sections of the survey (the HPV testing attitudes and beliefs items) (34), and excluded. Participants who were in the longest 2.5% and shortest 2.5% survey response times were also excluded.

For reporting descriptive socio-demographics, we calculated proportions, means (and standard deviations, SD), and used Pearson's Chi-Square test and *T*-tests to determine whether adequately screened and underscreened women differed significantly. We reported effect sizes using Cohen's d for *T*-tests, and Cramer's V for Pearson's Chi-Square test. We interpreted V = 0.1 as a small effect, V = 0.3 as a medium effect, and V = 0.5 as a large effect (35). BWS data was analyzed using the counting and the modeling approaches described by Aizaki and Fogarty (2019) (28). Corresponding to the counting approach, within each domain we calculated the best-minus-worst (BW) total score for each attribute and attribute level and used the function “bws2.count” from the package “support.BWS2” in R. Further, we used conditional logistic regression to estimate preferences and the marginal model that is based on the assumption that respondents evaluated all attribute-levels both when choosing the best and the worst attribute-level in each question (28). In each model (corresponding to Domain A and B), one attribute and one attribute-level (per attribute) were omitted from the utility function and treated as reference categories. Odds ratios (OR) and 95% confidence intervals (CI) were estimated using the “clogit” function and the “survival” package in R (36).

## Results

**Figure 1:**
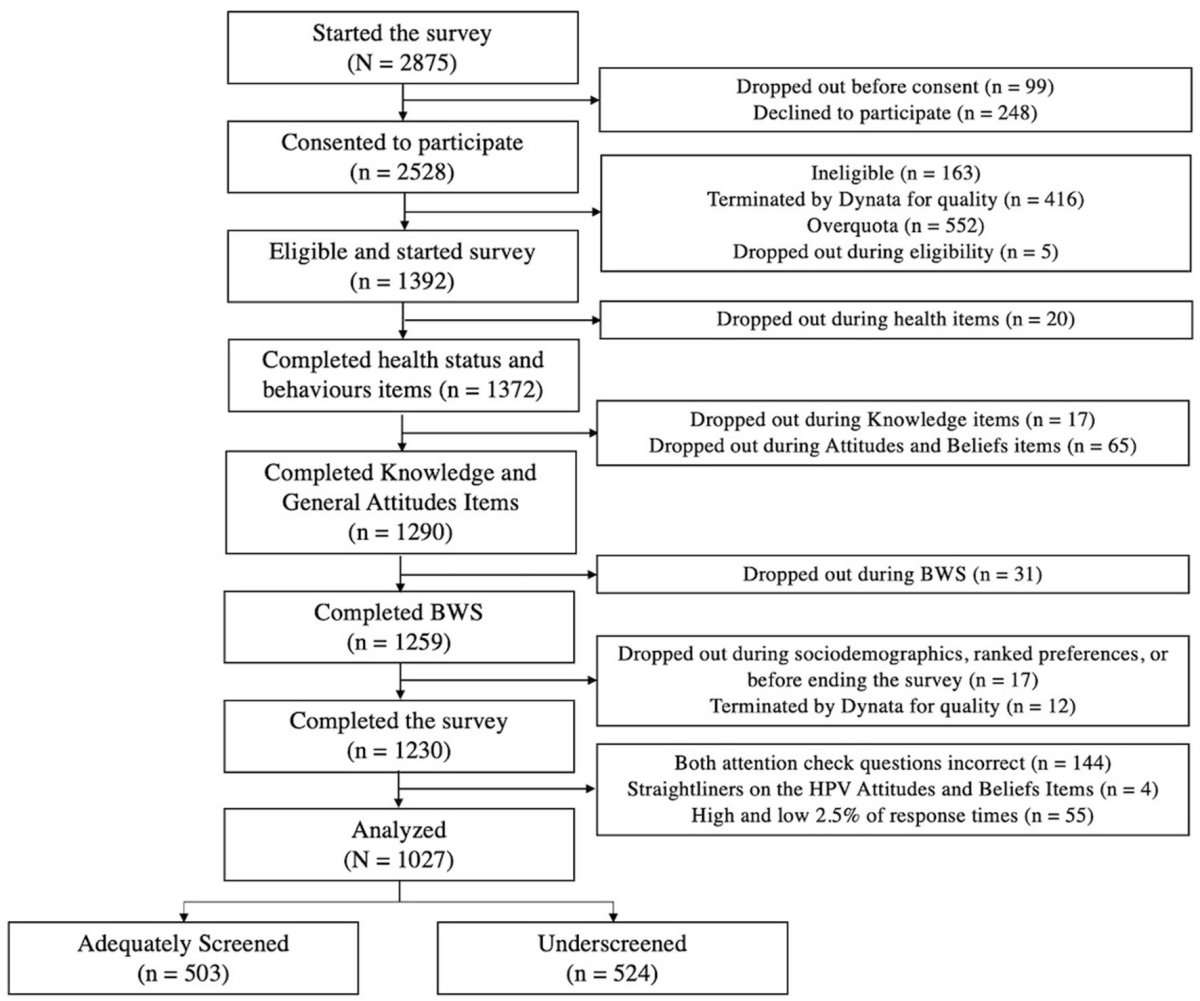
Participant flow.

A total of 1,230 participants completed the survey. Careless responders (*n* = 203) were identified and excluded from subsequent analyses. The final sample used for analyses consisted of 1,027 participants with *n* = 503 adequately screened and *n* = 524 underscreened (See Figure 1).

The sample consisted of mostly females (99.6%); the mean age was 48.4 years old; most spoke English at home (74.5%); most have lived in Canada for 10 years (96.4%); most had post-secondary education (69.9%); and most were married (59.5%). The following socio-demographic variables differed significantly between those who were adequately screened and those who were underscreened, although all effect sizes were small: ethnicity (χ^2^ = 11.03, *p* = 0.03, V = 0.10); self-perceived visible minority (χ^2^ = 10.65, *p* < 0.001, V = 0.10); language spoken at home (χ^2^ = 8.13, *p* = 0.02, V = 0.09); marital status (χ^2^ = 14.88, *p* < 0.001, V = 0.12); household income (χ^2^ = 30.48, *p* < 0.001, V = 0.17); and Canadian region (χ^2^ = 11.36, *p* < 0.001, V = 0.11) (See Table 1).

**Table 1:** Socio-demographic variables.

	**Total**	**Adequately screened**	**Underscreened**	**Between group difference**	**Effect size**
	***n*** = **1027**	***n*** = **503**	***n*** = **524**		
	***n*** **(%) or mean (SD)**	***n*** **(%) or mean (SD)**	***n*** **(%) or mean (SD)**		
**Age**	48.4 (12.6)	48.8 (12.0)	47.9 (13.1)	*p* = 0.28	d = 0.01
**Gender**					
Female	1023 (99.6)	501 (99.6)	522 (99.6)	χ^2^ = 0.00 *p* = 0.97	V = 0.00
Other	4 (0.4)	2 (0.4)	2 (0.4)		
**Ethnicity**					
North American Aboriginal	30 (2.9)	17 (3.4)	13 (2.5)	χ^2^ = 11.03 *p* = 0.03*	V = 0.10
Other North American	462 (45.0)	232 (46.1)	230 (43.9)		
European	340 (33.1)	176 (35.0)	164 (31.3)		
Asian	145 (14.1)	53 (10.5)	92 (17.6)		
Other	50 (4.9)	25 (5.0)	25 (4.8)		
**Self-perceived visible minority**					
Yes	198 (19.0)	75 (14.9)	120 (22.9)	χ^2^ = 10.65 *p*< 0.001*	V = 0.10
No	832 (81.0)	428 (85.1)	404 (77.1)		
**Influence of religion on health decisions**					
Yes	112 (10.9)	45 (8.9)	67 (12.8)	χ^2^ = 3.90 *p*= 0.05	V = 0.06
No	915 (89.1)	458 (91.1)	457 (87.2)		
**Language spoken at home**					
English	765 (74.5)	394 (78.3)	371 (70.8)	χ^2^ = 8.13 *p*= 0.02*	V = 0.09
French	211 (20.5)	90 (17.9)	121 (23.1)		
Other	51 (5.0)	19 (3.8)	32 (6.1)		
**Living in Canada for 10 years**					
Yes	990 (96.4)	490 (97.4)	500 (95.4)	χ^2^ = 2.94 *p*= 0.09	V = 0.05
No	37 (3.6)	13 (2.6)	24 (4.6)		
**Education (any post-secondary)**					
Yes	718 (69.9)	359 (71.4)	359 (68.5)	χ^2^ = 1.00 *p*= 0.32	V = 0.03
No	309 (30.1)	144 (28.6)	165 (31.5)		
**Marital status**					
Married/common-law	611 (59.5)	326 (64.8)	285 (54.4)	χ^2^ = 14.88 *p*< 0.001*	V = 0.12
Single	377 (36.7)	155 (30.8)	222 (42.2)		
Dating but do not live with partner	39 (3.8)	22 (4.4)	17 (3.2)		
**Household income in 2019 before the COVID-19 pandemic (CAD)**					
< 40,000	260 (25.3)	91 (18.1)	169 (32.3)	χ^2^ = 30.48 *p*< 0.001*	V = 0.17
40,000-79,999	354 (34.5)	179 (35.6)	175 (33.4)		
> 80,000	394 (38.4)	224 (44.5)	170 (32.4)		
Prefer not to answer	19 (1.9)	9 (1.8)	10 (1.9)		
**Canadian region**					
Western	314 (30.6)	157 (31.2)	157 (30.0)	χ^2^ = 11.36 *p*< 0.001*	V = 0.11
Central	652 (63.5)	304 (60.4)	348 (66.4)		
Eastern	61 (5.9)	42 (8.3)	19 (3.6)		

* Indicates a significant difference between adequately screened and underscreened groups.

### Preferences for screening methods and cervical cancer screening intervals

The analysis of preference for screening methods (attributes) shows that both adequately screened (_adq_) and underscreened women (_und_) preferred co-testing (BWs _adq_ = 977; BWs _und_ = 418) or the HPV test (BWs _adq_ = −224; BWs _und_ = −397) compared to the Pap test (BWs _adq_ = −333; BWs _und_ = −522). We found 100% and 51% higher odds of preference for co-testing vs. Pap in adequately and underscreened participants respectively. Importantly, among adequately screened women self-sampling was the least preferred screening method (BWs = −420) while in underscreened women, self-sampling was the most preferred screening method (BWs = 501) (See Table 2).

**Table 2:** Preferences for screening intervals (Domain A).

	**Whole sample** (*n* = 1027)		**Adequately screened** (*n* = 503)		**Underscreened** (*n* = 524)	
**Attributes**	**BWs**	**OR (95% CI)**	**BWs**	**OR (95% CI)**	**BWs**	**OR (95% CI)**
Pap Test	−855	ref	−333	ref	−522	ref
HPV Test	−621	1.06 (1.01; 1.10)	−224	1.06 (1.00; 1.13)	−397	1.06 (1.00; 1.12)
Pap and HPV test	1395	1.69 (1.62; 1.76)	977	2.00 (1.88; 2.14)	418	1.51 (1.43; 1.60)
HPV Self–Sampling	81	1.23 (1.17; 1.28)	−420	0.91 (0.85; 0.97)	501	1.56 (1.48; 1.66)
**Levels for attribute: Pap test**						
Every 3 years	305	1.75 (1.66; 1.84)	369	2.71 (2.51; 2.92)	−64	1.22 (1.14; 1.30)
Every 5 years	−108	ref	−44	ref	−64	ref
Every 10 years	−1052	0.49 (0.46; 0.51)	−658	0.31 (0.29; 0.33)	−394	0.68 (0.64; 0.73)
**Levels for attribute: HPV test**						
Every 3 years	341	1.65 (1.57; 1.73)	331	2.26 (2.10; 2.44)	10	1.28 (1.19; 1.36)
Every 5 years	−115	ref	−55	ref	−60	ref
Every 10 years	−847	0.54 (0.52; 0.57)	−500	0.39 (0.36; 0.42)	−347	0.69 (0.64; 0.73)
**Levels for attribute: Pap and HPV test**						
Every 3 years	1289	2.14 (2.03; 2.25)	948	3.64 (3.36; 3.94)	341	1.42 (1.33; 1.52)
Every 5 years	756	ref	443	ref	313	ref
Every 10 years	−650	0.36 (0.34; 0.38)	−414	0.22 (0.20; 0.24)	−236	0.52 (0.49; 0.56)
**Levels for attribute: HPV self–sampling**						
Every 3 years	585	1.68 (1.60; 1.77)	216	2.14(1.99; 2.31)	369	1.42 (1.33; 1.52)
Every 5 years	243	ref	−44	ref	287	ref
Every 10 years	−747	0.48 (0.46; 0.50)	−592	0.37 (0.35; 0.40)	−155	0.57 (0.53; 0.61)

Pertaining to screening intervals (attribute levels), for all screening methods and independent of screening status, women preferred most a 3-year interval followed by a 5-year interval and a 10-year interval between tests. For example, in adequately screened women we found BWs = 948; BWs = 443; and BWs = −414 for screening with both the Pap and HPV test every three; five; and ten years respectively. In both subgroups and for all screening methods, we found significantly higher preferences for screening every 3 years compared to 5 years and lower preferences for screening every 10 years vs. 5 years, e.g., in adequately screened women, OR = 3.64 (CI: 3.36; 3.94) and OR= 0.22 (CI = 0.20; 0.24) respectively (See Table 2).

### Preferences for screening methods and age of initiation for cervical cancer screening

In domain B, the most preferred screening method (attributes) in both subgroups was co-testing (BWS _adq_ = 1207; BWs _und_ = 508) and preferences were significantly higher compared to the Pap test (OR _adq_ = 2.20; CI: 2.06; 2.35; OR _und_ = 1.59; CI: 1.50; 1.69). As with the results for domain A (screening intervals) the least preferred screening method in adequately screened women was self-sampling (BWs = −693) and preferences were 20% lower compared to Pap (OR= 0.80; CI: 0.75; 0.85). In underscreened women, self-sampling was the second most preferred option (BWs= 378) after co-testing and preferences were significantly higher for self-sampling compared to Pap (OR = 1.50; CI: 1.41; 1.59) (See Table 3).

**Table 3:** Preferences for age of screening initiation (Domain B).

	**Whole sample** (*n* = 1027)		**Adequately screened** *(n* = 503)		**Underscreened** (*n* = 524)	
**Attributes**	**BWs**	**OR (95% CI)**	**BWs**	**OR (95% CI)**	**BWs**	**OR (95% CI)**
Pap Test	−870	ref	−320	ref	−550	ref
HPV Test	−530	1.08 (1.04; 1.13)	−194	1.06 (1.00; 1.13)	−336	1.10 (1.04; 1.16)
Pap and HPV test	1715	1.82 (1.74; 1.90)	1207	2.20 (2.06; 2.35)	508	1.59 (1.50; 1.69)
HPV Self–Sampling	−315	1.12 (1.08; 1.17)	−693	0.80 (0.75; 0.85)	378	1.50 (1.41; 1.59)
**Levels for attribute: Pap test**						
21 years old	296	1.73 (1.65; 1.82)	305	2.33 (2.16; 2.50)	−9	1.36 (1.27; 1.46)
25 years old	−214	ref	−94	ref	−120	ref
30 years old	−952	0.54 (0.51; 0.56)	−531	0.41 (0.38; 0.44)	−421	0.66 (0.62; 0.71)
**Levels for attribute: HPV test**						
21 years old	421	1.72 (1.64; 1.81)	354	2.31 (2.14; 2.48)	67	1.36 (1.27; 1.46)
25 years old	−131	1.07 (1.01; 1.12)	−69	1.06 (0.98; 1.14)	−62	1.10 (1.03; 1.17)
30 years old	−820	ref	−479	ref	−341	ref
**Levels for attribute: Pap and HPV test**						
21 years old	1372	2.09 (1.99; 2.20)	961	3.17 (2.93; 3.43)	411	1.52 (1.42; 1.63)
25 years old	777	1.20 (1.14; 1.26)	497	1.19 (1.10; 1.28)	280	1.21 (1.13; 1.29)
30 years old	−434	ref	−251	ref	−183	ref
**Levels for attribute: HPV self–sampling**						
21 years old	387	1.58 (1.50; 1.66)	94	1.98 (1.84; 2.13)	293	1.34 (1.25; 1.43)
25 years old	37	1.15 (1.10; 1.21)	−158	1.17 (1.09; 1.26)	195	1.13 (1.06; 1.21)
30 years old	−739	ref	−629	ref	−110	ref

For all screening methods and independent of screening status, women preferred screening initiation (attribute levels) at age 21, followed by 25, and 30. With the exception of screening with HPV testing starting at 25 years in adequately screened women (where the result was not statistically significant [OR = 1.06; CI: 0.98; 1.14]), independent of the screening method, preferences were significantly higher for screening initiation at age 21 or 25 compared to 30 years (See Table 3).

## Discussion

Using BWS methodology, this study aimed to understand Canadian women's current preferences toward cervical cancer screening intervals, ages of screening initiation, and screening methods.

We found that the three-year screening option was most preferred by women regardless of screening status and this aligns with findings from women in the HPV FOCAL trial where acceptance of extending screening intervals to 4–5 years was found to be lower than expected (54%), especially as participants received educational content about these changes (19, 20). This has been identified as a common concern for women in other countries where screening programs have extended screening intervals in the context of the implementation of HPV-based primary screening. For instance, several studies in Australia and England suggest that many women believe that longer intervals could result in delayed detection and treatment of cervical cancer (21, 37–39). Our results strongly suggest that extending screening intervals beyond the current 3-year practices will be of concern to Canadian women. When Canadian provinces introduce HPV-based screening with extended screening intervals, specific educational content addressing reasons for and reassuring women about the safety and appropriateness of these extended intervals will be essential to ensure broad acceptance. Waller et al. (40) demonstrated that providing messages about HPV test accuracy, safety, and explaining the speed of cell change in cervical cancer development resulted in more positive attitudes toward extending screening intervals to 5 years. Although two or three-year intervals are currently implemented in Canada (41), some healthcare providers recommend screening more often (15), which may further reinforce resistance to screening intervals being extended beyond 3 years.

Regarding age of initiation, we found that both adequately screened and underscreened women significantly preferred 21 years and 25 years for screening initiation, in contrast to the findings of Smith et al. (19) who found that 69% of women accepted commencing screening with the HPV test at 30 years (19). Importantly, participants in the FOCAL trial were provided education about and experience with HPV based screening, whereas our study participants only received minimal information to complete the survey. Therefore, differences in acceptance likely emanate from the differences in education and highlight the need for large-scale efforts on a population level to educate women about these changes. Additionally, as using BWS methodology allows us to understand women's preferences while considering other alternatives, our findings might suggest that women are less likely to endorse later screening initiation when comparing it to earlier screening ages than examining it in isolation. Public health authorities should consider such trade-offs women face with these policy changes when planning the implementation and communication of changes. For example, a study of Australian women aged 18–24 who are no longer recommended for cervical cancer screening found that those who had previously received a Pap test felt less positive about the extended age of initiation than those who had never received a Pap test, suggesting a need to specifically consider the concerns of women directly impacted by policy changes (42). Importantly, results of qualitative studies align with our results as they have highlighted concern from women about younger women being at risk of developing cervical cancer when considering screening initiation beginning at age 25 (21, 37, 38).

Our results showed that adequately screened women preferred to be screened with co-testing or the HPV test compared to the Pap test, while underscreened women preferred HPV self-sampling or co-testing. The contrast in the preferred methods between adequately and underscreened women indicates that offering them different sample collection methods (something that cytology-based screening does not currently allow) may increase uptake of cervical cancer screening, especially amongst underscreened women. As suggested by Vahabi & Lofters. (18, 43), offering HPV self-sampling, which underscreened women significantly preferred over the Pap test in our study, could be an effective method of screening to reach this population in Canada. In Canada, studies have found that difficulties with scheduling an appointment, inconvenient clinic hours, lack of time, and social stigma associated with screening to be barriers faced by underscreened women (23, 44). Studies from Australia and New Zealand, among others, have found that offering HPV self-sampling can increase screening participation amongst underscreened women (45–47), and our findings indicate that its use would be worth exploring in Canada as well.

### Limitations and implications

Our study provides an initial signal that without adequate education and communication from health authorities describing the rationale for extended intervals and increased age of screening initiation with HPV-based screening, Canadian women may be reluctant to accept these changes. To confirm our findings, larger nationally representative samples would be needed. Our study results can inform future investigations of women's preferences for cervical cancer screening, as well as inform public health communications surrounding potential policy changes. As Canada transitions to HPV-based screening, it is essential that public health authorities adequately inform women about the rationale for, and advantages of extended screening intervals and increased age of screening initiation.

## Data availability statement

The raw data supporting the conclusions of this article will be made available by the authors, without undue reservation.

## Ethics statement

This study received ethical approval from the Research Ethics Board of The Integrated Health and Social Services University Network West-Central Montreal (CIUSSS-CO) (Project ID: 2021-2632). The patients/participants provided their written informed consent to participate in this study.

## Author contributions

PZ and OT completed data analysis and wrote the first draft of the manuscript. BH, GG-M, LS, JB, GO, and ZR provided critical feedback on manuscript revisions. ZR supervised all stages of the project. All authors had substantial contribution in study conceptualization and design. All authors approved the final manuscript.

## Funding

This work was supported by the Canadian Institutes of Health Research (CIHR) Project Grant #165905. OT is supported by the Canadian Institutes of Health Research (CIHR)-Frederick Banting and Charles Best Doctoral award (Award No. FBD-170837) outside of the submitted work.

## Conflict of interest

JB's employer, the Australian Centre for the Prevention of Cervical Cancer, has received donated tests from HPV test manufacturers for research and validation studies. The remaining authors declares that the research was conducted in the absence of any commercial or financial relationships that could be construed as a potential conflict of interest.

## Publisher's note

All claims expressed in this article are solely those of the authors and do not necessarily represent those of their affiliated organizations, or those of the publisher, the editors and the reviewers. Any product that may be evaluated in this article, or claim that may be made by its manufacturer, is not guaranteed or endorsed by the publisher.
