# Supplementary material for: Assessing Canadian women's preferences for cervical cancer screening: A brief report
Source: Front Public Health. 2022 Jul 28;10:962039. doi: 10.3389/fpubh.2022.962039 (PMC9366717; doi:10.3389/fpubh.2022.962039)
Supplement: Supplementary file 1 [file Data_Sheet_1.pdf]

Appendix A. Informative Statements

Figure 1. Informative statement about cervical cancer screening

Please read carefully the following information about cervical cancer screening:

Cervical cancer is the fourth most common cancer in women worldwide. The **cervix** is the lower part of the uterus (womb). The cervix connects the uterus to the vagina (birth canal).

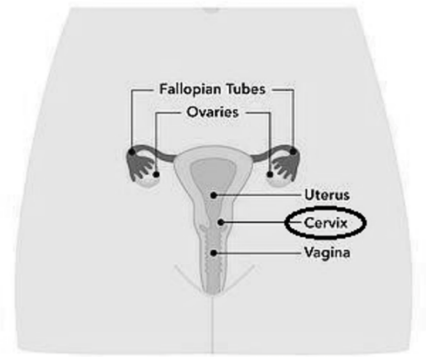

The **Pap test** (also called Pap smear) has been used to prevent cervical cancer for decades. During a **Pap tes**t, a healthcare professional uses a brush to gently scrape cells from your cervix. A new test called the **Human Papillomavirus (HPV) test** is also now available to prevent cervical cancer.

Image adapted from: Gouvernement du Québec (https://www.quebec.ca/en/health/health-issues/cancer/cervical-cancer)

Figure 2. Informative statement about HPV testing

Please carefully read the following information about HPV testing:

Human papillomavirus (HPV) is the most common sexually transmitted infection. Almost all cervical cancers are caused by HPV. Cervical cancer is a highly preventable disease.

The Pap Test

- Cervical cells are looked at in the lab under a microscope to check for abnormal cells
- Currently used for routine cervical cancer screening in Canada
  - Every 2 or 3 years (starting at age 21 or 25, continuing until 65 or 70 depending on the province/territory in which you live)

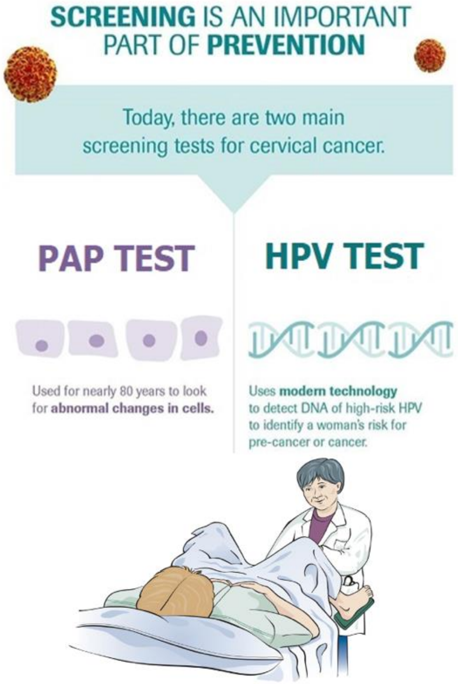

The HPV Test

- Uses specialized technology to look for the presence of HPV DNA
- **Not yet used** for routine cervical cancer screening in Canada
  - Public health authorities are in the process of developing programs to introduce it
- Research shows that if HPV DNA is not found, women are at very low risk for cervical cancer and do not need to screen for cervical cancer as often as with the Pap test (e.g., every 5 years)

For both tests, the procedure to collect the cell sample is the same (see picture above)

Images adapted from: Roche Diagnostics (https://twitter.com/CxCaDx/status/897066598560870400?s=20&t=icp5Yxm5IESnEuHVc-6HKw)
CancerCare Manitoba (https://www.cancercare.mb.ca/screening/cervix)

Figure 3. Informative statement about HPV self-sampling

Please carefully read the following information about HPV self-sampling:

The HPV test detects the presence of HPV DNA in cervical cells. The collection of the cells for the HPV test can be done by your healthcare professional.

The collection of the cells can also be done by you in privacy. This is called **HPV self-sampling**, where you insert a thin swab into the vagina to collect cervical cells using a kit approved by Health Canada. The sample is then sent to a laboratory for analysis. The results are then returned to your clinic/healthcare provider, who will communicate with you. HPV self-sampling is **not yet used** but is being considered for routine cervical cancer screening in Canada. See the figure containing a description of HPV self-sampling in four steps.

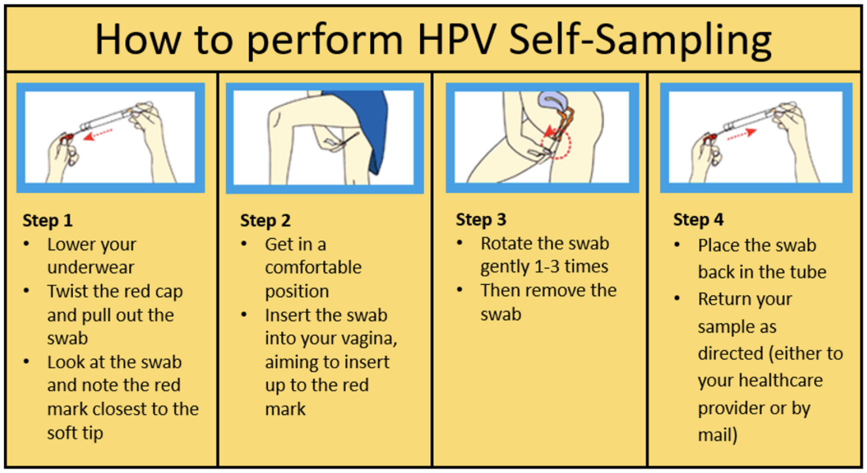

Image adapted from: The Royal Australian College of General Practitioners (https://www1.racgp.org.au/newsgp/gp-opinion/self-collection-of-hpv-samples-a-guide-for-gps)

Figure 4. Informative statement about cervical cancer screening options

Please carefully read the following information about cervical cancer screening options:

You have been presented with several methods for cervical cancer screening. Here is a reminder of what each of these methods are:

	**Pap test**	**HPV test**	
**Type of Analysis**	Cervical cells are looked at under a microscope to check for abnormal cells	Uses specialized technology to look for the presence of HPV DNA in cervical cells	
**Sample collection**	Done by a healthcare professional in a clinic/office	**Option 1**<br>Done by a healthcare professional in a clinic/office	**Option 2**<br>Self-sampling done by yourself in privacy using a kit approved by Health Canada

Imagine you are offered a choice among these methods by your healthcare professional. In this last set of questions, we want to know which of these methods you would prefer.
