# Supplementary material for: Assessing Canadian women's preferences for cervical cancer screening: A brief report
Source: Front Public Health. 2022 Jul 28;10:962039. doi: 10.3389/fpubh.2022.962039 (PMC9366717; doi:10.3389/fpubh.2022.962039)
Supplement: Supplementary file 2 [file Data_Sheet_2.pdf]

<u>Appendix B. Best-Worst Scaling Questions</u>

Preferences

In this next set of questions, you will be asked to select two out of four different situations: 1) **your least preferred** situation and 2) **your most preferred** situation. There should be two marks, <u>one mark next to your most preferred situation</u>, and <u>one mark next to your least preferred situation.</u>

Please see the sample question below:

Sample question		
LEAST preferred	Options	MOST preferred
X	I would prefer to watch movies once per week	
	I would prefer to play tennis two days per week	
	I would prefer to go shopping three times per week	
	I would prefer to go to the gym every day	X

In summary, you would have selected “I prefer to go to the gym every day” as your most preferred item, and “I prefer to watch movies once per week” as your least preferred item.

Please follow the same instructions for answering the next set of questions.

Domain A. Screening intervals

For each of the following nine questions, use the same strategy as in the example above to select **one** situation that you prefer **least** AND **one** situation that you prefer **most.** The questions may seem repetitive, but the last part of each sentence is different (where it says "every X years").

Question 1		
LEAST preferred	Options	MOST preferred
	Cervical cancer screening with the **Pap test** every 3 years	
	Cervical cancer screening with the **HPV test** every 3 years	
	Cervical cancer screening with **both the Pap test and the HPV test** every 3 years	
	Cervical cancer screening with the **HPV test using HPV self-sampling** every 3 years	

Question 2		
LEAST preferred	Options	MOST preferred
	Cervical cancer screening with the **Pap test** every 3 years	
	Cervical cancer screening with the **HPV test** every 5 years	
	Cervical cancer screening with **both the Pap test and the HPV test** every 10 years	
	Cervical cancer screening with the **HPV test using HPV self-sampling** every 5 years	

Question 3		
LEAST preferred	Options	MOST preferred
	Cervical cancer screening with the **Pap test** every 3 years	
	Cervical cancer screening with the **HPV test** every 10 years	
	Cervical cancer screening with **both the Pap test and the HPV test** every 5 years	
	Cervical cancer screening with the **HPV test using HPV self-sampling** every 10 years	

Question 4		
LEAST preferred	Options	MOST preferred
	Cervical cancer screening with the **Pap test** every 5 years	
	Cervical cancer screening with the **HPV test** every 3 years	
	Cervical cancer screening with **both the Pap test and the HPV test** every 10 years	
	Cervical cancer screening with the **HPV test using HPV self-sampling** every 10 years	

Question 5		
LEAST preferred	Options	MOST preferred
	Cervical cancer screening with the **Pap test** every 5 years	
	Cervical cancer screening with the **HPV test** every 5 years	
	Cervical cancer screening with **both the Pap test and the HPV test** every 5 years	
	Cervical cancer screening with the **HPV test using HPV self-sampling** every 3 years	

Question 6		
LEAST preferred	Options	MOST preferred
	Cervical cancer screening with the **Pap test** every 5 years	
	Cervical cancer screening with the **HPV test** every 10 years	
	Cervical cancer screening with **both the Pap test and the HPV test** every 3 years	
	Cervical cancer screening with the **HPV test using HPV self-sampling** every 5 years	

Question 7		
LEAST preferred	Options	MOST preferred
	Cervical cancer screening with the **Pap test** every 10 years	
	Cervical cancer screening with the **HPV test** every 3 years	
	Cervical cancer screening with **both the Pap test and the HPV test** every 5 years	
	Cervical cancer screening with the **HPV test using HPV self-sampling** every 5 years	

Question 8		
LEAST preferred	Options	MOST preferred
	Cervical cancer screening with the **Pap test** every 10 years	
	Cervical cancer screening with the **HPV test** every 5 years	
	Cervical cancer screening with **both the Pap test and the HPV test** every 3 years	
	Cervical cancer screening with the **HPV test using HPV self-sampling** every 10 years	

Question 9		
LEAST preferred	Options	MOST preferred
	Cervical cancer screening with the **Pap test** every 10 years	
	Cervical cancer screening with the **HPV test** every 10 years	
	Cervical cancer screening with **both the Pap test and the HPV test** every 10 years	
	Cervical cancer screening with the **HPV test using HPV self-sampling** every 3 years	

<u>Domain B. Age of screening initiation</u>

In this next set of questions, you will once again be asked to select two out of four different situations: 1) **your least preferred** situation and 2) **your most preferred** situation. There should be two marks, <u>one mark next to your most preferred situation</u>, and <u>one mark next to your least preferred situation.</u>

For each of the following nine questions, please select **one** statement that you prefer **<u>least</u>** AND **one** statement that you prefer **<u>most.</u>** The questions may seem repetitive, but the last part of each sentence is different (where it says "starting at X years old").

Question 1		
LEAST preferred	Options	MOST preferred
	Cervical cancer screening with the **Pap test** starting at age 21 years old	
	Cervical cancer screening with the **HPV test** starting at age 21 years old	
	Cervical cancer screening with **both the Pap test and the HPV test** starting at age 21 years old	
	Cervical cancer screening with the **HPV test using HPV self-sampling** starting at age 21 years old	

Question 2		
LEAST preferred	Options	MOST preferred
	Cervical cancer screening with the **Pap test** starting at age 21 years old	
	Cervical cancer screening with the **HPV test** starting at age 25 years old	
	Cervical cancer screening with **both the Pap test and the HPV test** starting at age 30 years old	
	Cervical cancer screening with the **HPV test using HPV self-sampling** starting at age 25 years old	

Question 3		
LEAST preferred	Options	MOST preferred
	Cervical cancer screening with the **Pap test** starting at age 21 years old	
	Cervical cancer screening with the **HPV test** starting at age 30 years old	
	Cervical cancer screening with **both the Pap test and the HPV test** starting at age 25 years old	
	Cervical cancer screening with the **HPV test using HPV self-sampling** starting at age 30 years old	

Question 4		
LEAST preferred	Options	MOST preferred
	Cervical cancer screening with the **Pap test** starting at age 25 years old	
	Cervical cancer screening with the **HPV test** starting at age 21 years old	
	Cervical cancer screening with **both the Pap test and the HPV test** starting at age 30 years old	
	Cervical cancer screening with the **HPV test using HPV self-sampling** starting at age 30 years old	

Question 5		
LEAST preferred	Options	MOST preferred
	Cervical cancer screening with the **Pap test** starting at age 25 years old	
	Cervical cancer screening with the **HPV test** starting at age 25 years old	
	Cervical cancer screening with **both the Pap test and the HPV test** starting at age 25 years old	
	Cervical cancer screening with the **HPV test using HPV self-sampling** starting at age 21 years old	

Question 6		
LEAST preferred	Options	MOST preferred
	Cervical cancer screening with the **Pap test** starting at age 25 years old	
	Cervical cancer screening with the **HPV test** starting at age 30 years old	
	Cervical cancer screening with **both the Pap test and the HPV test** starting at age 21 years old	
	Cervical cancer screening with the **HPV test using HPV self-sampling** starting at age 25 years old	

Question 7		
LEAST preferred	Options	MOST preferred
	Cervical cancer screening with the **Pap test** starting at age 30 years old	
	Cervical cancer screening with the **HPV test** starting at age 21 years old	
	Cervical cancer screening with **both the Pap test and the HPV test** starting at age 25 years old	
	Cervical cancer screening with the **HPV test using HPV self-sampling** starting at age 25 years old	

Question 8		
LEAST preferred	Options	MOST preferred
	Cervical cancer screening with the **Pap test** starting at age 30 years old	
	Cervical cancer screening with the **HPV test** starting at age 25 years old	
	Cervical cancer screening with **both the Pap test and the HPV test** starting at age 21 years old	
	Cervical cancer screening with the **HPV test using HPV self-sampling** starting at age 30 years old	

Question 9		
LEAST preferred	Options	MOST preferred
	Cervical cancer screening with the **Pap test** starting at age 30 years old	
	Cervical cancer screening with the **HPV test** starting at age 30 years old	
	Cervical cancer screening with **both the Pap test and the HPV test** starting at age 30 years old	
	Cervical cancer screening with the **HPV test using HPV self-sampling** starting at age 21 years old	
